# Bacterial Diversity Associated with *Cinachyra cavernosa* and *Haliclona pigmentifera*, Cohabiting Sponges in the Coral Reef Ecosystem of Gulf of Mannar, Southeast Coast of India

**DOI:** 10.1371/journal.pone.0123222

**Published:** 2015-05-04

**Authors:** C. Jasmin, Abdulaziz Anas, Shanta Nair

**Affiliations:** 1 Council of Scientific and Industrial Research (CSIR)—National Institute of Oceanography (NIO), Regional Centre, Cochin, Kerala, 682018, India; 2 Council of Scientific and Industrial Research (CSIR)—National Institute of Oceanography (NIO), Dona Paula, Goa, 403004, India; CAS, CHINA

## Abstract

Sponges are abundant, diverse and functionally important organisms of coral reef ecosystems. Sponge-associated microorganisms have been receiving greater attention because of their significant contribution to sponge biomass, biogeochemical cycles and biotechnological potentials. However, our understanding of the sponge microbiome is limited to a few species of sponges from restricted geographical locations. Here, we report for the first time the bacterial diversity of two cohabiting sponges, viz. *Cinachyra cavernosa* and *Haliclona pigmentifera*, as well as that in the ambient water from the coral reef ecosystems of the Gulf of Mannar, located along the southeast coast of India. Two hundred and fifty two clones in the 16S rRNA gene library of these sponges were grouped into eight distinct phyla, of which four belonged to the core group that are associated only with sponges. Phylogenetic analysis of the core bacteria showed close affinity to other sponge-associated bacteria from different geographical locations. γ-Proteobacteria, Chloroflexi, Planctomycetes and Deferribacter were the core groups in *C*. *cavernosa* while β and δ-Proteobacteria performed this role in *H*. *pigmentifera*. We observed greater OTU diversity for *C*. *cavernosa* (H^ǀ^ 2.07) compared to H. pigmentifera (H^ǀ^ 1.97). UniFrac analysis confirmed the difference in bacterial diversity of the two sponge species and also between the sponges and the reef water (p<0.001). The results of our study restate the existence of a host driven force in shaping the sponge microbiome.

## Introduction

Sponges are primitive members of the evolutionary tree, accounting for 8500 taxonomically validated species [1,2]. Soest et al.[2] lucidly reviewed the diversity and global distribution of sponges. Sponges are the vital link between benthic and pelagic coupling in coral reef ecosystems, with varied functional roles ranging from biogeochemical cycling of nutrients to facilitating primary production and eroding the carbonate reef structure [3,4]. Many of these diverse metabolic functions are strongly supported by the associated microorganisms [5–7] that comprise up to 40% of the total tissue volume of sponges, a density several orders of magnitude higher than that of the surrounding seawater. Therefore, understanding the diversity of microorganisms associated with sponges is necessary to puzzle out the functioning of the complex coral reef ecosystem, especially in the energy coupling between the benthic and pelagic communities [8].To date, 12 candidate phyla and 2 archaeal lineages have been identified from sponges from the Mediterranean and Pacific regions [9],but little is known from the Indian waters. These phyla include Chloroflexi (formerly green non-sulfur bacteria), Acidobacteria, Actinobacteria, and *α*-, *γ*-, and *δ*-proteobacteria [9]. Studies on the community structure of sponge-associated bacteria from different geographical regions are essential in order to understand the ecological implications of sponge—microbe interactions.

Initially sponge microbiomes were classified into three ecological groups, viz. generalists, associates and specialists [10], and later based on molecular analysis of high throughput sequence data (~32000 tag sequences)the terminology was changed to variable, core and species specific [11,12]. However, both these terminolgies of classifications are being used in the literature. Generalists or variables are those bacteria that are retrieved both from sponges and seawater and their diversity may vary among the sponges depending on the environmental conditions. Sponge-associated or core bacteria are those which are associated only with sponges regardless of species or geographically separated regions [13]. The existence of a sponge-associated or core group of microorganisms was comprehensively explored recently, by performing phylogenetic analysis of all publicly available rRNA gene sequences originated from sponges [14,15]. These studies suggested the role of host-driven factors, such as metabolite exchange, in shaping the core group. Such relationships have been predicted by Reiswig [16] and a number of examples to support this hypothesis are available, including the translocation of glucose from symbiotic green algae to their sponge host [17] and translocation of nitrogen from bacteria to the sponge [18]. The specialists or species-specific groups are endemic microorganisms that are restricted to a single species of sponge irrespective of its geographic locations and are transmitted vertically from the progenitor. However, massive studies from geographically distinct locations are imperative for confirming the existence of variable, core and species-specific populations in sponges.

The coral reef ecosystem in the Gulf of Mannar (GoM), located along the southeast tip of the Indian coast, has received considerable research attention and is also the first Marine Biosphere Reserve in India due to its ecological importance and vulnerability of biological resources [19]. It is also known as the hot spot of sponge diversity and approximately 275 species of sponges have been reported from here [20].Earlier studies on sponges from Indian waters were mainly dealing with their response to pollution or the biotechnological potentials of the cultivable bacteria associated with them [19,21]. In this study we report for the first time the bacterial diversity associated with two cohabiting sponges in GoM, viz. *Cinachyra cavernosa* and *Haliclona pigmentifera*, and compare with that in the ambient water, based on a culture-independent 16S rRNA gene library approach.

## Materials and Methods

### Ethics statement

The field collections carried out for the purpose of this paper did not involve endangered or protected species. Permission was received from Principal Chief Conservator of Forests and Chief Wildlife Warden for entering coral reef ecosystems of GoM. No specific permission was required to collect the analyzed sponge samples.

### Sample collection and Treatment

**Fig 1:**
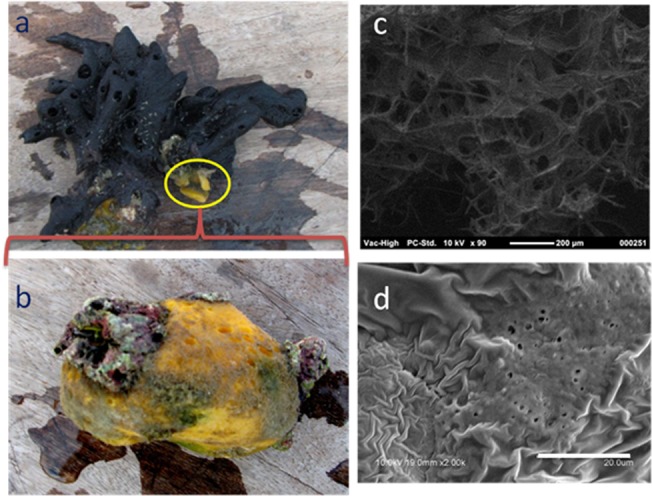
Normal (a & b) and Scanning Electron microscope image (c& d) of *Haliclona pigmentifera* (a & c) and *Cinachyra cavernosa* (b&d). Scale bars for SEM micrographs: c = 200 μm and d = 20 μm

We collected two species of sponges, viz. *H*. *pigmentifera* and *C*. *cavernosa*, the latter was attached to the first one, from the coral reef ecosystems of GoM by scuba diving from 5–10 m depth, and species identities were confirmed by microscopic observation of morphological characters. *H*. *pigmentifera* belongs to the Phylum: Porifera, Class: Demospongiae, Order: Haplosclerida, Family: Chalinidae. Its body is soft with prominent openings on its finger like branches and is black in color (Fig 1A). *C*. *cavernosa* belongs to the Phylum: Porifera, Class: Demospongiae, Order: Spirophorida, Family: Tetillidae. Its spherical body has unevenly arranged golf ball like depressions and is bright yellow in color with occasional grayish brown and green patches (Fig 1B). In order to minimize contamination, nitrile gloves were worn while handling the samples. Sponge specimens were washed thoroughly with calcium–magnesium-free artificial seawater (CMF-ASW, pH 7.2) to remove residual sand, debris and loosely attached microorganisms. The washed sponge samples were cut into 1cm pieces, transferred into sterile polythene bags and stored in liquid nitrogen. Reef water samples were collected close to where the sponges were collected. Water samples (~3000 ml) were passed through 0.2 μ polycarbonate membrane filters (Millipore) andthe filters were preserved in liquid nitrogen. Sponge samples and the filter papers stored in liquid nitrogen were transported to the laboratory for analysis.

### Scanning Electron Microscopy

The surface layer of each sponge sample was sliced, dehydrated and placed on a microscope sample holder and gold sputtering was done in an argon atmosphere. Adequate care was taken to obtain a homogenous cell gold coating.The surfaces of *H*. *pigmentifera* and *C*. *cavernosa* were imaged on a Neoscope JCM 5000 scanning electron microscope (JEOL, Japan).

### DNA extraction from sponges and water samples

Genomic DNA was extracted from sponge tissue following Ouyang et al.[22] with slight modifications. Briefly, 100 mg sponge tissue was macerated with 400 μl lysis buffer (0.5 M NaCl, 100 mM EDTA, 10 mM Tris pH 8.0), and incubated with lysozyme (15 mgml^-1^) for 1 hr at 37°C. Subsequently, SDS (1%) and proteinase K (500 μgml^-1^) were added to the solution and continued the incubation for 2 hr at 55°C. Genomic DNA was extracted with Phenol:Chloroform:isoamyl alcohol (25:24:1), followed by chloroform: isoamyl alcohol (24:1) twice. Genomic DNA in the aqueous phase was precipitated with 0.6 volume isopropanol and washed copiously with 70% ethanol. The DNA pellet was air dried, dissolved in TE buffer and stored at -20°C.

Genomic DNA was extracted from the filters following Boström et al [23]with slight modifications. Briefly, 0.2 μ polycarbonate membrane filters (Millipore) were incubated at 37°C for 1 hr in lysis buffer (NaCl 400 mM, Sucrose 750 mM, EDTA 20 mM and Tris HCl 50 mM) containing 1 mgml^-1^ lysozyme. Subsequently, SDS (1%) and proteinase K (100 μgml^-1^) were added to the solution and continued incubation for 5 hr at 55°C. Further 0.6 volume of isopropanol was added and the DNA was precipitated by keeping at -20°C for 60 min. The DNA pellet was washed with 70% ethanol, dissolved in TE buffer and stored at -20°C until used. Integrity of the isolated DNA were assessed on 0.8% agarose gel, and the purity was analyzed spectrophotometrically by measuring the ratio of absorbance at 260/280 nm in a ND-1000 spectrophotometer (NanoDrop, Thermo Scientific).

### Amplification of bacterial 16S rRNA gene

The 16S rRNA gene of bacteria was amplified using universal primers [27F:AGAGTTTGATC(AC)TGGCTCAG and 1492R: GGTTACCTTGTTACGACTT] [24] in a 25 μl reaction volume containing 1 μl DNA sample (50–100 ng), 1 μl each of primers (10 picomoles μl^-1^), 2.5 μl 10X Taq polymerase buffer (NEB, Canada), 0.5U Taq DNA polymerase (NEB, Canada) and 200 μM each dNTPs (NEB, Canada). The cycling conditions were as follows: initial denaturation at 95°C for 2 min, followed by 30 cycles of denaturation at 95°C for 40 sec, annealing at 55°C for 40 sec, extension at 72°C for 1.5 min and a final extension for 10 min at 72°C.

### Construction of 16S rRNA gene libraries and sequencing

PCR products were purified using Nucleopore Genetix brand Sure Extract PCR clean up/ Gel extraction kit (Genetix Biotech, India) and cloned into pGEM-T Easy vector (Promega, USA), following the supplier’s manual and transformed into chemically competent *E*.*coli* DH5αby heat shock method. The recombinant colonies were picked up and grown at 37°C for overnight in LB broth, an aliquote of which was preserved in glycerol at -80°C and the remaining was used for plasmid preperation. Recombinant plasmids were purified using Nucleopore Genetix brand SureSpin plasmid mini prep kit (Genetix Biotech, India), and used as a template for sequencing PCR reactions in combination with vector specific primers, T7 and SP6, and gene specific internal primers 1090R [GCTCGTTGCGGGACTTAACC] [25] and 515R [ATTACCGCGGCTGCTGG] [26]. Sequencing PCR was done with ABI PRISM BigDye terminator ready reaction mix (Life Technologies, USA). The cycle extension products were purified following ethanol/EDTA/sodium acetate precipitation. The products were analyzed on an Applied Biosystems ABI 3730xl DNA analyzer.

### Phylogenetic and statistical analysis

The sequences were edited using the software Sequencher V4.10.1 (GeneCodes Corporation, Ann Arbor, MI, USA) and checked for the presence of any contaminating vector sequences using the Vecscreen program of NCBI. The chimeric sequences detected using DECIPHER (http://decipher.cee.wisc.edu/FindChimeras.html) [27] were removed from the data set, and the final sequences were classified into different phyla using RDP classifier (http://rdp.cme.msu.edu/index.jsp) [28]. Sequences were trimmed at 5′end at Bact341 and 3′ at Bact534 and grouped using PSI algorithm to calculate the operational taxonomic units (OTUs), rarefaction analysis, Shannon-Wiener and Chao1 indices using the online program Fastgroup II (http://fastgroup.sdsu.edu/) at a clustering threshold of 97% sequence identity [29]. Rarefaction was performed to estimate OTU richness as a function of the number of clones sequenced and to determine whether the total diversity in the samples was well represented by the number of clones sequenced in each library. Rarefaction curves were plotted using Origin 7.5 (OriginLab Corporation, USA). Sequences from all OTUs were searched in NCBI using BLASTn and the nearest neighbors were selected. Sequences were multiple aligned using clustalW and neighbor-joining and maximum-likelihood phylogenetic trees were constructed using MEGA 5.2 version [30]. Bootstrap tests were performed 1000 times using MEGA 5.2.

The phylogenetic tree of OTUs were analyzed using the online multivariate statistic software package Unifrac to compare the diversity [31]. Transformed sequence data were used to perform multivariant cluster analysis and principal component analysis (PCA) using PRIMER v.6 software package (Plymouth Marine Laboratory) to understand the influence of bacteria on structuring the diversity in sponges.

### Nucleotide accession numbers

The GenBank accession numbers for the bacterial sequences are KF373120- KF373212 for *H*. *pigmentifera*, KC861009-KC86167 for *C*. *cavernosa* and KC878327-KC878385 and KF036053- KF036081 for reef waters.

## Results

The scanning electron microscopic images showed large pores (~100 μm diameter) on the surface of *H*. *pigmentifera* (Fig 1C), while the pores were small (~2 μm diameter) on *C*. *cavernosa* (Fig 1D). The number of clones obtained from reef water, *H*. *pigmentifera* and *C*. *cavernosa* were 89, 93 and 159, respectively. These sequences were de-replicated into 34, 19 and 27 OTUs for reef water, *H*. *pigmentifera* and *C*. *cavernosa*, respectively. Rarefaction showed no instance where the curves reached clear saturation, indicating that further sampling of clone libraries, would have revealed additional diversity (S1 Fig). However, the rarefaction curves were steeper, indicating greater potential diversity in the bacterial community of these samples. Shannon-Wiener and Chao1 indices showed that the observed bacterial diversity was higher in reef water compared to sponges, and was in the order of reef water >*C*. *cavernosa* >*H*. *pigmentifera* (Table 1).

**Table 1:** Statistical analysis of clone library.

	*C*. *cavernosa*	*H*. *pigmentifera*	Reef water
**No of clones sequenced**	159	*93*	89
**Number of OTUs**	27.0	*19*.*0*	34.0
**Shannon- Wiener index**	2.07	*1*.*97*	2.79
**Chao 1**	45.75	*43*.*0*	138.17

**Fig 2:**
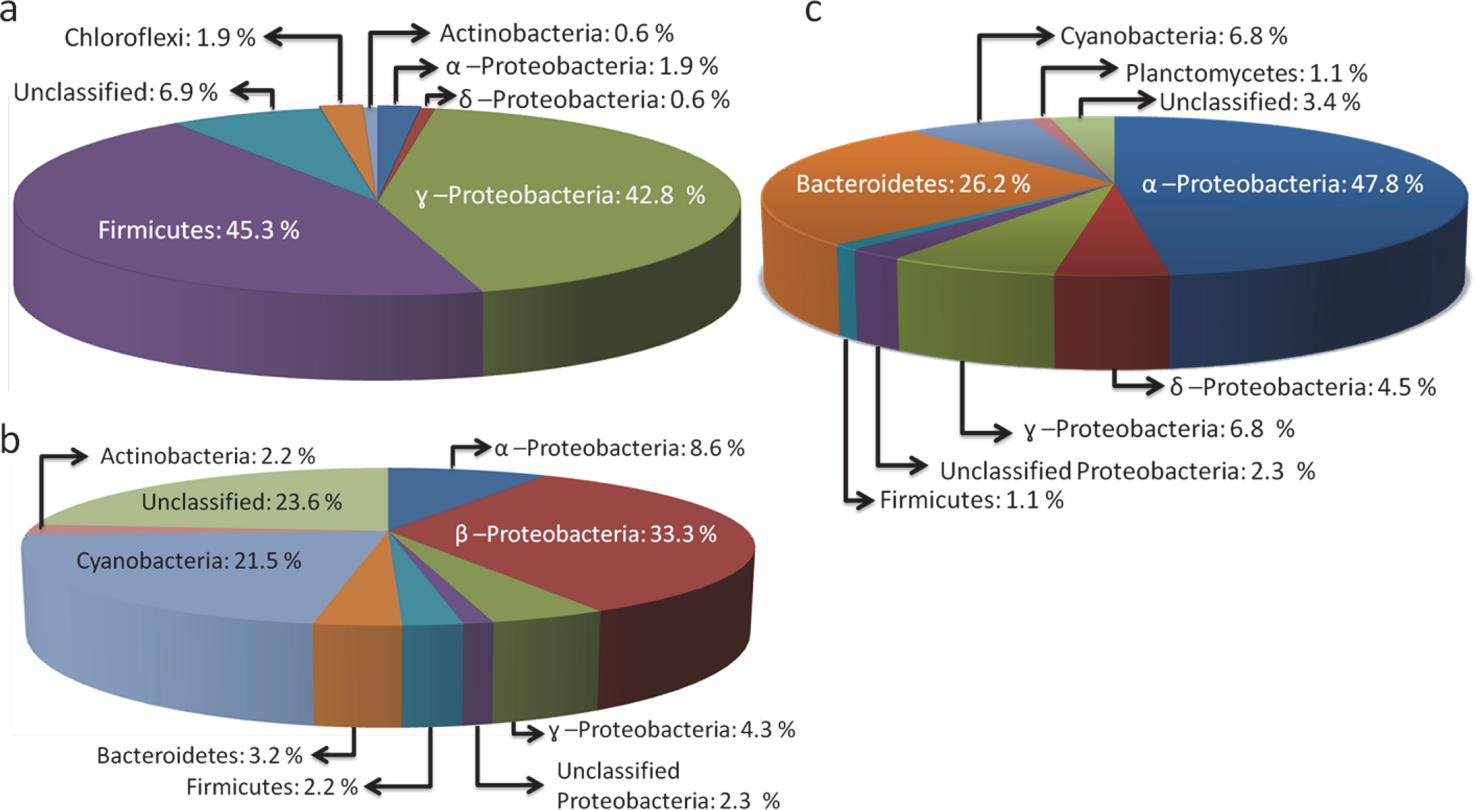
Pi chart illustrating the community structure of bacteria associated with *Cinachyra cavernosa* (a), *Haliclona pigmentifera* (b) and reef water (c).

There was a clear difference in the bacterial diversity associated with the two sponge species cohabiting in GoM and *in situ* reef water based on clone library analysis (Fig 2). The dominant bacterial phylum in *C*. *cavernosa* was Firmicutes (45.3%) followed by γ-Proteobacteria (42.8%), whereas in *H*. *pigmentifera* it was β-Proteobacteria (33.3%) followed by Cyanobacteria (21.5%). Phylum Chloroflexi (1.9%) and Actinobacteria (0.6%) were the minor groups in *C*. *cavernosa* (Fig 2A). A major share of clones from *H*. *pigmentifera* (23.6%) was clustered to unidentified bacteria (Fig 2B). Interestingly, the clone library of water samples was different from the sponge clone library (Fig 2C). There was a dominance of α-proteobacteria (47.8%) followed by Bacteroidetes (26.2%) in the clone library of water samples collected from the proximity of sponge samples (Fig 2C).

**Fig 3:**
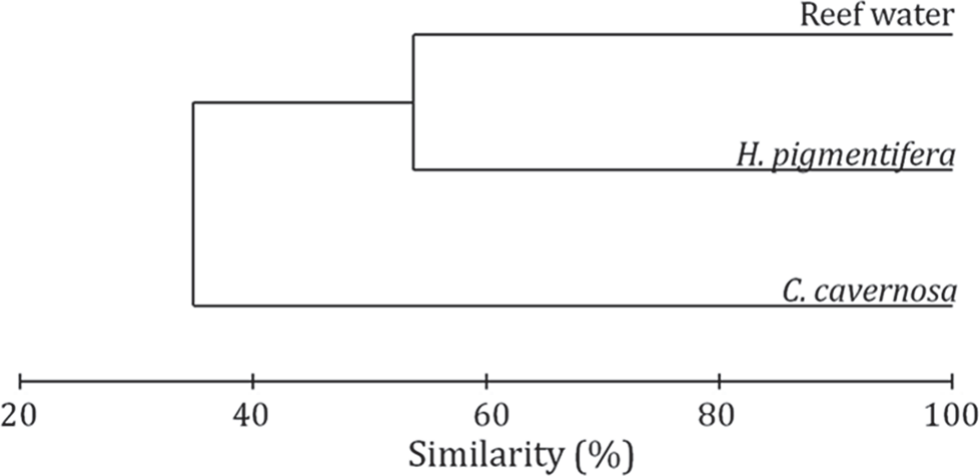
Dendrogram of cluster analysis showing similarities in percent of OTUs of 16S rRNA gene sequences obtained from sponge associated microorganisms and reef water.

**Fig 4:**
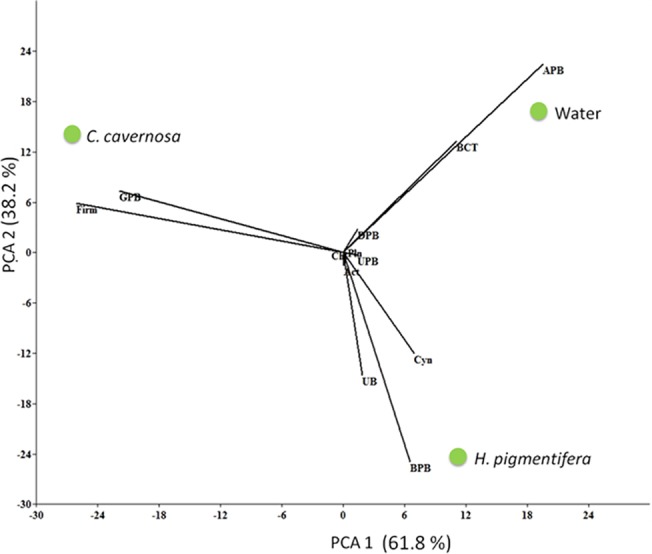
PCA ordination biplots of bacterial diversity of *C*. *cavernosa*, *H*. *pigmentifera* and Reef water. The bacterial groups (is it clade or group) are represented as γ- proteobacteria (GPB), Firmicutes (Firm), β-Proteobacteria (BPB), Bacteroidetes (BCT), Planctomycete (Pln), Chloroflexi (Chl), Actinobacteria (Act), Unclassified bacteria (UB), Unclassified proteobacteria (UPB), Cyanobacteria (Cyn).

The dissimilarity between the bacterial communities of *C*. *cavernosa* and reef water was evident in the cluster analysis of OTUs (Fig 3). Bacterial diversity in *H*. *pigmentifera* was more similar to that of reef water with a similarity of more than 50%, while the bacterial diversity in *C*. *cavernosa* formed a different cluster. The Unifrac analysis showed that the differences in diversity of bacteria between *C*. *cavernosa* and reef water were statistically significant (p<0.001), while the differences between *H*. *pigmentifera* and water were not significant. PCA ordination based on bacterial community structure in different samples showed that *C*. *cavernosa* and *H*. *pigmentifera* were influenced by different bacterial communities and these were different from that of the reef water (Fig 4). PCA ordination plot showed that the microbial community structure of *C*. *cavernosa* was positively influenced by Firmicutes and γ-Proteobacteria while in *H*. *pigmentifera* it was Cyanobacteria, β-Proteobacteria and unclassified bacteria. In the reef water the microbial community was influenced by Bacteroidetes and α-Proteobacteria.

**Fig 5:**
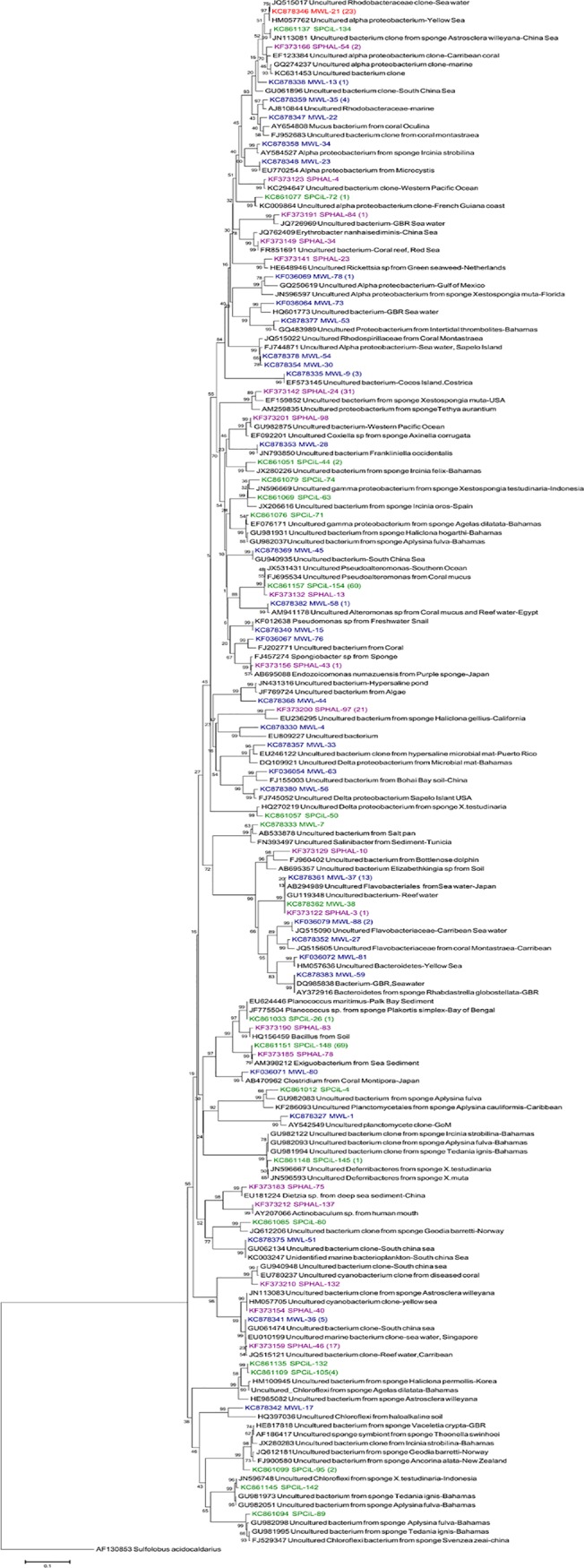
Rooted neighbor-joining phylogenetic tree based on 16S rRNA gene sequences retrieved from the clone library of the sponge *C*. *cavernosa* (green), *H*. *pigmentifera* (purple) and reef water (red). The numbers at the nodes are percentages indicating the levels of bootstrap support based on a neighbor joining analysis of 1,000 resampled data sets. Scale bar represents 10% estimated sequence divergence. *Sulfolobus acidocaldarius* was used as an out group. Numbers in bracket indicates the additional clones of the same OTU present in the library.

The phylogenetic tree of OTUs associated with *C*. *cavernosa* (Fig 5 and S2 Fig) showed that the *Exiguobacterium* sp is the most prominent OTU under the class Firmicutes. In Phylum Proteobacteria, γ class was more prominent with 67 clones, grouped into 6 OTUs. Among the γ- Proteobacteria, 59 clones showed close similarity to uncultured *Pseudoalteromonas* of marine origin. Five OTUs of *Chloroflexi* sp (11 clones) also were observed in *C*. *cavernosa*. Minor representations of Planctomycetes (1 OTU), Deferribacter (2 OTU), and Actinobacteria (1 OTU) were also found in the clone library of *C*. *cavernosa*. Clone library of bacteria associated with *H*. *pigmentifera* was represented by β-Proteobacteria (32 clones represented by single OTU), δ-Proteobacteria (22 clones represented by single OTU), Cyanobacteria (20 clones represented by 3 OTUs) and α —Proteobacteria (8 clones represented by 5 OTUs) (Fig 5). Minor representations of Firmicutes (2 clones represented by 2 OTUs), Actinobacteria (2 clones represented by 2 OTUs), Bacteroidetes (3 clones represented by 2 OTUs) and γ-Proteobacteria (4 clones represented by 3 OTUs) were also observed. The clone library of the reef water sample consisted of α-Proteobacteria (44 clones under 12 OTUs) and Bacteroidetes (22 clones under 7 OTUs). Cyanobacteria (6 clones under 1 OTU) and 5 OTUs each of γ-Proteobacteria (6 clones) and δ- Proteobacteria (5 clones) were also present. Minor representations of Firmicutes, Chloroflexi, Planctomycetes and unclassified bacteria (single clone represented by single OTU) were also seen.

## Discussion

The present study compares the diversity of bacteria associated with cohabiting sponges, *C*. *cavernosa* and *H*. *pigmentifera*. It was observed (during scuba diving) that *C*. *cavernosa* was attached on the surface of *H*. *pigmentifera*, and such an association is rarely reported in sponges and this would be very appropriate for studying the role of host driven forces in shaping sponge microbiome. The cohabitation of *Haliclona* sp with other sponge *Geodia* sp was reported earlier from Mexican Pacific coast [32]. To the best of our knowledge the microbial symbionts from such closely associated sponges are not reported to date. The associated microorganisms were grouped into core or variable categories based on the similarity of 16S rRNA gene sequences obtained with sponge derived sequences available in NCBI. Only those organisms with sequence similarity with sponge-derived sequences were tagged as core group, while others were considered as generalists. Since no previous reports on bacterial diversity of *C*. *cavernosa* and *H*. *pigmentifera* are available for comparison, we could not assign any bacteria as species-specific to the two host sponge species studied. Members of Proteobacteria, Planctomycetes, Chloroflexi and Deferribacteres in *C*. *cavernosa* and Proteobacteria in *H*. *pigmentifera* were grouped as core bacteria, while all others were variables. Diversity index and number of OTUs were higher in *C*. *cavernosa* compared to *H*. *pigmentifera*. The bacterial community of *H*. *pigmentifera* was more than 50% similar to that of reef water whereas that of *C*. *cavernosa* formed a different cluster. Recently Cuvelier et al [33] reported the microbial communities associated with sponge *Cinachyrella* sp from South Florida reef using 16S rRNA gene tag pyrosequencing, and identified Proteobacteria as prominent group. Studies from the Mediterranean and Pacific regions have shown that the closely related sponge hosts may have similar microbial diversity irrespective of their geographic differences, while different species of sponges living in the same environment may not share the same microbial diversity [13].Our study restates the previous reports that the two cohabiting sponges of different species have different bacterial signatures.

Although Proteobacteria was recorded as a major group both in sponges and water, significant difference among sub groups were evident. Dominance of this phylum is unexceptional as they are known to be ubiquitous in marine environment and exists both as planktonic and in association with organisms [34]. Previous studies have reported the occurrence of Proteobacteria as associates in sponges regardless of their geographical locations, such as *Haliclona* sp. from Monterey harbor in USA [5] and Great Barrier reef in Australia [1], *Aplysina cavernicola* from the coast of Elba in the Mediterranean Sea [35] and *Banyuls-sur-Mer* on the coast of Marseille in France [36], *Rhopaloeides odorabile* from Davies reef in Australia[6], *Theonella swinhoei* from western Caroline islands in the Republic of Palau [37], *Halichondria panicea* from the Adriatic Sea (Croatia), the north sea near Helgoland (Germany), The Baltic sea near Kiel (Germany) [38] and *Stelletta tenui*, *Halichondria* sp, *Dysidea avara* and *Craniella autraliensis* from South China sea [39]. They have the advantage of producing extracellular enzymes and thereby contributing to biogeochemical cycles. In sponges they have varied functions such as nitrogen fixing, manipulating host reproduction and supporting sponge defense mechanism [1,37,39]. The rRNA sequence of four OTU (5 clones) of γ-Proteobacteria in the clone library of *C*. *cavernosa* had high similarity with uncultured γ-Proteobacteria of sponges from different geographical locations. One OTU (2 clones) in the clone library of *H*. *pigmentifera* also showed high similarity with γ-Proteobacteria of sponges. These γ-Proteobacteria could be considered as a core group of *C*. *cavernosa* and *H*. *pigmentifera* following Hentschel et al [13]. The sequence analyses showed that the dominant OTU need not form the core group in sponges. For instance, 61 clones of *Pseudoalteromonas* sp represented by two OTU were a variable group associated with *C*. *cavernosa* and their nearest neighbors were observed in seawater from different geographical locations.

β-Proteobacteria was dominant in the clone library (32 clones) of *H*. *pigmentifera* and was represented by a single OTU. This OTU is a putative sponge associate with its nearest neighbors present in *Tethya aurantium*, *Xestospongia muta* and *Haliclona gellius* [5,40]. β-Proteobacteria were not observed in the clone library of *C*. *cavernosa* and reef water. α-Proteobacteria were dominant in the reef water and the representatives of this class in sponges were variables. However, high prevalence of α-Proteobacteria has been reported from many sponge species of distinct geographical locations [41]. δ-Proteobacteria were under represented in the clone library of *C*. *cavernosa*, and the single OTU observed may be a core bacteria. Interestingly, 23.6% of clones in *H*. *pigmentifera* library classified as unclassified bacteria by RDP classifier showed close similarity with δ-Proteobacteria in NCBI BLAST analysis. This OTU could be classified as a core group considering its high sequence similarity with δ-Proteobacteria from sponges, *H*. *gellius*, *X*.*muta* and *Crella cyathophora* [5,42,43].Our results show that different subclasses of Proteobacteria present both in *C*. *cavernosa* and *H*. *pigmentifera* occur either as core or generalist group.

Chloroflexi was found only in the clone library of *C*. *cavernosa*. Eleven clones in the library of *C*. *cavernosa* were clustered into five OTUs and these can be presumed as core group strictly belonging to sponge specific cluster. Low abundance of Chloroflexi has been reported recently from *Cinachyrella* in South Florida reef [33]. Not surprisingly, the single Chloroflexi OTU found in the reef water did not fall into sponge specific cluster. Chloroflexi is largely an uncharacterized group of bacterial phyla associated with a wide variety of marine sponges, with many sponge specific clusters identified [44–47]. Chloroflexi might play important roles in sponge nutrition and defense [45], and has the ability to fix atmospheric carbon through photosynthesis in shallow waters and thus may provide carbonaceous compounds to the sponge host [48].

Firmicutes was observed as a dominant phylum in *C*. *cavernosa* (45. 3%) while it had very low representation in *H*. *pigmentifera* (2.2%) and in reef water (1.1%). The major OTU representing Firmicutes belonged to *Exiguobacterium* sp. The *Exiguobacterium* sp has been reported widely from marine environments [49], yet their presence in sponges has not been recorded. The nearest neighbors of *Exiguobacterium* sp present in the clone library of *C*. *cavernosa* have been reported from sediment and water samples, indicating their generalist nature. Many Firmicutes have been reported as major bacterial population associated with polar and cold-temperate marine sponges [50,51], however the core groups are uncommon [10,50]. One OTU each of Planctomycete (1 clone) and Deferribacteres (2 clone) also was recorded as core bacteria of *C*. *cavernosa* in our study. Planctomycetes were ubiquitous in marine environment and have been reported to occur in association with sponges [52]. The localization of planctomycetes within the sponge mesohyl matrix has been demonstrated using fluorescent *insitu* hybridization [53]. These organisms may be playing a key functional role in nutrient recycling and carbon and sulphur cycles in the marine systems. Single Planctomycete OTU found in the reef water did not fall into sponge specific cluster. Deferribacteres were found only in the clone library of *C*. *cavernosa* and they clustered with core microorganisms of sponges reported from different geographical locations [41,43]. Deferribacteres have been reported to play functional role in sulfur cycles in oxygen minimum environments [54].

Cyanobacteria were dominant in the clone library of *H*. *pigmentifera* (21. 5%) and reef water (6.8%), but were absent in *C*. *cavernosa*. Cyanobacteria in *H*. *pigmentifera* were generalists as their nearest neighbors were found in the clone library of reef water. Interestingly, 18 out of 20 clones of Cyanobacteria in the clone library of *H*. *pigmentifera* were represented by a single OTU and its sequence had high similarity with a cyanobacterial clone in reef water. Cyanobacteria have been reported from many sponges including *Haliclona* sp and are known to provide a range of specialized services for host’s survival and growth, including photosynthesis, nitrogen fixation, UV protection and antifedants [55]. Also cyanobionts contribute up to 80% of sponge’s carbon budget through photosynthesis or phagocytosis and digestion of symbiotic microbes [55]. However, our study indicates that cyanobacteria in *H*. *pigmentifera* were generalists, which colonized in sponge tissue from reef water.

Overall, *C*. *cavernosa* had higher OTU diversity and core group of bacteria compared to *H*. *pigmentifera*. Proteobacteria, Chloroflexi, Planctomycetes and Deferribacter were observed as core groups of *C*. *cavernosa* while only β and δ-Proteobacteria were found as core groups of *H*. *pigmentifera*. PCA analysis showed that the generalist bacteria such as Firmicutes and γ- Proteobacteria in *C*. *cavernosa* and Cyanobacteria and β-Proteobacteria in *H*. *pigmentifera* also had positive impact on the community structure of the respective sponge species. This may be due to different reasons ranging from its own physiology, feeding habits, and influence of immediate benthic environment as well as the water quality characteristics. The interaction between the associated microorganisms, such as antagonism and symbiosis also may influence the microbial community structure of sponges. However, further studies are required to confirm the role of these factors in influencing the diversity of bacteria associated with sponges. Nonetheless, the difference observed in the bacterial diversity between *C*. *cavernosa* and *H*. *pigmentifera* may be attributed to the difference in their pore size and canal systems. Large sized pores are evident in the SEM image of *H*. *pigmentifera*, which may facilitate continuous pumping of water at low pressure, leading to the enrichment of seawater bacterial flora. On the other hand, small sized pores, dense mesohyl and complicated canal systems in *C*. *cavernosa*, may induce pumping of water at high pressure and hence sponge specific clusters may get enriched [56]. The pumping activity retains the oxygenation of sponge mesohyl matrix, and it is easily conceivable that the reduction in pumping efficiency influences the microbial diversity [57]. The narrow canal systems, dense mesohyl and high bacterial abundance are considered as the characteristics of high microbial abundance sponges (HMA), while sponges qualified as low microbial abundance (LMA) have wide canal systems and lower abundance of microorganisms [58].

The present study gives the first insight into the bacterial diversity of cohabiting sponges living in the coral reef ecosystems of GoM. In short, our study reiterates that each species of sponge maintains signature microbial diversity regardless of their close proximity. *C*. *cavernosa* was dominated by Firmicutes and Proteobacteria with Proteobacteria, Planctomycetes, Chloroflexi and Deferribacter as core group. Cyanobacteria and β-Proteobacteria were dominant in *H*. *pigmentifera*, with β- proteobacteria being the sole core group. Future research may be focused on understanding the microbiome of cohabiting sponges from different geographic locations, which may give more insight into the role of host driven forces in shaping sponge microbiome.

## Supporting Information

S1 Fig Rarefaction curves for clone libraries fromseawater (A), *C*. *cavernosa* (B) and *H*. *pigmentifera* (C). (TIF)

S2 Fig Rooted maximum-likelihood phylogenetic tree based on 16S rRNA gene sequences retrieved from the clone library of the sponge *C*. *cavernosa* (green), *H*. *pigmentifera* (brown) and reef water (blue). The numbers at the nodes are percentages indicating the levels of bootstrap support based on a neighbor joining analysis of 1,000 resampled data sets. Scale bar represents 10% estimated sequence divergence. *Sulfolobus acidocaldarius* was used as an out group. Numbers in bracket indicates the additional clones of the same OTU present in the library.
(TIF)
