# Supplementary figures and images for: Bacterial Diversity Associated with Cinachyra cavernosa and Haliclona pigmentifera, Cohabiting Sponges in the Coral Reef Ecosystem of Gulf of Mannar, Southeast Coast of India
Source: PLoS One. 2015 May 4;10(5):e0123222. doi: 10.1371/journal.pone.0123222 (PMC4418615; doi:10.1371/journal.pone.0123222)

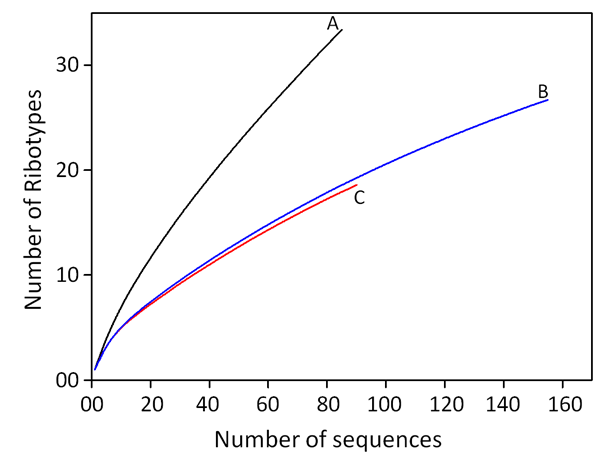

Supplement: S1 Fig — (TIF) [file pone.0123222.s001.tif]
